# Melanoma: From Incurable Beast to a Curable Bet. The Success of Immunotherapy

**DOI:** 10.3389/fonc.2015.00152

**Published:** 2015-07-13

**Authors:** Maria Libera Ascierto, Ignacio Melero, Paolo Antonio Ascierto

**Affiliations:** ^1^Department of Oncology, Johns Hopkins University, Baltimore, MD, USA; ^2^Department of Oncology, Centro de Investigación Médica Aplicada (CIMA), Clinica Universidad de Navarra, Pamplona, Spain; ^3^Unit of Melanoma, Cancer Immunotherapy and Innovative Therapy, Istituto Nazionale Tumori Fondazione “G. Pascale”, Napoli, Italy

**Keywords:** immunotherapy, melanoma, adoptive T cell therapy, immunomodulatory antibodies, vaccination, combination therapy

## Abstract

After Coley’s observation in 1891 of tumor regression in a patient who developed a postoperative infection, the field of immunotherapy is finally reborn. Avoiding immune destruction is now considered a hallmark of cancer, and the immunotherapy arena has exploded with the recent advances demonstrating an improvement in survival and a durability of response in patients with different cancer types, which translates into improved overall survival benefit. Here, we provide an overview of the main immune-oncology treatment strategies that, either alone or in combination, are undergoing clinical development. Namely, we will refer to those immunotherapeutic strategies that include adoptive transfer of *ex vivo* activated T cells, immunomodulatory monoclonal antibodies, and cancer vaccines. Our major focus will be to describe these approaches in melanoma, a cancer type transformed by immunotherapy into a potentially curable disease.

## Introduction

It was at the end of the nineteenth century when, following Coley’s observation of a spontaneous tumor regression due to postsurgical fever (1), the role played by immune system against cancer development came out of the shadow. Indeed, the longtime underestimated Coley’s observation has recently inspired the development of numerous anti-cancer immune approaches, which led to the rebirth of immunotherapy as epitomized by immunotherapy’s designation as the year breakthrough of 2013 by Science magazine (2). In the past 20 years, clinical and pathological observations have emphasized even more that the roles played by host immune cells in the tumor stroma are critical determinants of cancer biology and key factors for the success or failure of cancer therapy. Such discoveries have changed the field of tumor immunology so that “avoiding immune destruction” is now considered an emerging hallmark of cancer (3).

Although both innate (natural killer cells, macrophages, and dendritic cells) and adaptive T cell-mediated immunity arms are postulated to play coordinated roles in cancer immune surveillance (4–6), most emphasis in immunotherapy has been placed on the adaptive immune response. In support of the critical role of T lymphocytes in human against cancer, tumor-infiltrating T lymphocyte cell (TIL) density estimated by tissue immunohistochemical (IHC) analysis has revealed a positive prognostic association between high density of CD8+ effector memory cells and overall survival (OS) of patients with melanoma and other cancer types (7, 8). Analysis conducted on pretreatment melanoma biopsies using gene expression analysis revealed that activation of interferon (IFN) signal transduction pathway signified by phosphorylated STAT1, IFNγ, CCR5 CXCR3, and their ligands are positively correlated with favorable clinic outcome in breast cancer in terms of response to therapy (9). Furthermore, colon, ovary, melanoma, and breast cancer specimens have shown a positive correlation between up regulation of genes involved in the CD4+ Th1 adaptive immune response and a more favorable prognosis (5, 10).

To date, there is limited evidence with regard to the antigen specificity and understanding of the mechanisms of such spontaneous TIL cells. However, these observations have given rise to a conceptual model in which an adaptive T cell response composed of both cytotoxic CD8+ T cells (CTLs) and CD4+ Th1 cells control cancer progression. According to this immunosurveillance interpretation, the ability to produce cytokines, such as IFN-γ and tumor necrosis factor (TNF)-α, together with the expansion and activation of cytotoxic CD8+ T cells, is considered critical (11). Perhaps, because of these observations, attempts to exploit CTLs, TILs, and CD4+ Th1 cells have been the predominant immunotherapeutic approaches for cancer.

Here, we provide an overview of the most successful strategies in immunotherapy, used either as single agents or in synergistic treatment combinations. These include adoptive transfer of *ex vivo* activated T cells, immunomodulatory monoclonal antibodies (mAbs), and cancer vaccines. Challenges, advantages, and disadvantages associated with each of these approaches are discussed. Our major focus will be to describe these approaches in melanoma, a cancer type well known for its resistance to conventional radio and chemotherapy. However, since 2011, there have been considerable advances in melanoma management with the approval of several treatments. In this context, the most encouraging results in stabilizing the disease and increasing the OS have been obtained by using immunotherapeutic approaches (12), which transformed the once unbeatable melanoma beast into a curable bet.

## Adoptive T Cell Therapy

Adoptive cellular immunotherapy involves administering auto­logous or allogeneic tumor-reactive T or NK cells to patients in order to achieve tumor regression. Today, adoptive T therapy represents one of the most promising therapies in the field of cancer treatment, showing extremely promising results in patients with B-cell leukemias lymphomas, and also in melanoma (13–15). Adoptive T cell therapy involves the isolation of lymphocytes with high affinity for tumor antigens, which can be selected *ex vivo*, stimulated, expanded, and infused back into the patient. The feasibility of generating T cells *ex vivo* is limited by the initial frequency of tumor antigen-specific T cells, which can be very low. Initially, tumor-specific T cells were selected from peripheral blood mononucleated cell (PBMCs) and cloned by limiting dilution in the presence of irradiated feeder cells. However, cultures of monoclonal and polyclonal T cells do show much efficiency neither in T cell expansion nor at inducing tumor rejection. The advance in tumor-specific T cells has been generated from enriched sources, such as TILs populations and tumor-draining lymph nodes. Before TIL infusions, patients must be heavily preconditioned by total body irradiation and lymphodepleting chemotherapy, which is supposed to eliminate both regulatory T cells and the competition for homeostatic cytokines, which sustain T-cell proliferation and survival. In melanoma, it has been shown that numerous tumor antigen-specific T cells can be isolated from excised material of a tumor mass, dissociating cells into single cell suspensions and adding the T-cell growth factor interleukin-2 (IL-2) (16). Several clinical trials using this approach have provided highly promising results, especially in melanoma. For instance, a series of studies collectively involving 93 patients with stage IV melanoma were treated with the adoptive transfer of autologous TILs administered in conjunction with IL-2 following a lymphodepleting preparative regimen on three clinical trials. Objective-response rates in the three trials using lymphodepleting preparative regimens (chemotherapy alone or with 2 or 12 Gy total body irradiation) were 49, 52, and 72%, respectively (15). Of particular note, 22% of patients had a complete tumor regression and most of these patients have been alive and disease-free for longer than 8 years (16). Encouraging results were also shown in another study by treating 57 patients with IV stage melanoma with unselected/young TIL and high-dose IL-2 following non-myeloablative lymphodepletion. Overall response rates (ORR) were observed up to 40% with 23 patients achieving complete or partial remission (17).

Despite the successes of T adoptive therapy with TILs, a clinical response is still not guaranteed for all patients. Indeed, not all patients respond to this type of therapy in the same way and the reasons behind this differential response to TIL T cell transfer remain unknown. It has been shown that tumors escape TIL transfer by several mechanisms and there is considerable evidence that TILs are blocked *in vivo* by many immunosuppressive molecules, such as programed cell death protein-1 (PD-1) and cytotoxic T cells antigen-4 (CTLA-4). Furthermore, increased expression of molecules with immunosuppressive properties, such as nitric-oxide synthase 1 (NOS1) (18) or indoleamine 2,3-dioxygenase (IDO) might block TIL activity in tumors and decrease response to adoptive T cell transfer therapy (8). As such, a clear portrait of the phenomena associated with the lack of response observed in some patients is necessary in order to prevent both costly regimens and life-threatening side effects associated with the therapy.

As a consequence of the immune repertoire selection that takes place in immunosuppressive tumors *in vivo*, the affinity of the repertoire of endogenous T cell receptor (TCRs) for tumor-specific antigens is suboptimal. Thus, several approaches have been developed to genetically engineer T cells with high affinity TCRs and thereby confer strong effector functions upon recognition of tumor-associated antigens. High avidity human TCRs are isolated either from *in vitro* cultures of naïve T cells with allogeneic peptide-pulsed antigen-presenting cells (19) or following vaccination of humanized mice expressing transgenic human leukocyte antigen (HLA) alleles together with the human TCR alpha and beta loci mutated to avoid mispairing with the endogenous murine TCR chains (20). Candidate TCRs are sequenced, cloned, and inserted into retroviral or lentiviral vectors, which can be then used to transduce autologous T cells from other patients with matching HLA restriction elements (21). The specificity of the TCR-transduced T cells is not altered compared to the parental T cell clone (22). Another problem associated with the transfer of genetically modified TCRs is that the recognition is restricted by a given HLA allele. This limitation is overcome by the use of chimeric antigen receptors (CARs) in which a single-chain antibody (artificially linked light and heavy chains) is coupled to the transmembrane and cytoplasmic signaling domains of the TCR complex (CD3-zeta) and co-stimulatory molecules (CD137 or CD28), that elicit lymphocyte activation upon antigen encounter. Due to the reduction of B cells numbers and B-cell aplasia following anti-CD19 CAR T cell treatment, this approach is taking the front seat of cancer treatments especially in hematologic malignancies (14, 23). Genetic modification of T cells can also involve inserting genes to improve the efficacy of the T cells or induce co-stimulation and inhibit apoptosis of modified T cells. Risks associated with modifying T cells include the emergence of serious toxicity, such as cytokine release syndrome (16) or organ damage due to overexpression of the recognized antigen or cross-reactivity with other self-antigens. However, safety can be improved by identifying those antigens that can be targeted to destroy cancer cells without toxic effects in normal tissues and by gene transfer of drug-inducible suicide genes to abrogate toxicity.

## Monoclonal Antibodies for Cancer Therapy

The use of mAbs in cancer therapy is one of the greatest clinical successes of the past decade. The fact that these antibodies can be used in the effective treatment of cancer is based on many years of comprehensive research into their complex structure, physical, chemical, and biological properties, specificity to targeted antigens, and therapeutic activity *in vivo*, either alone or in combination. Following the discovery of mABs by Cesar Miltein and George Kohler, their therapeutic use has relied on strategies to humanize their mouse sequences in such a way that their immune potential is much reduced when administered to humans.

In cancer therapy, an antibody can impact tumor regression through various mechanisms. First, direct action of the antibody on tumor cells through binding to a tumor cell surface receptor can induce receptor activation or an antagonist activity leading to tumor cell apoptosis. Second, the antibody may not act directly on the tumor but instead induce an immune response, which mediates tumor cell death by phagocytosis, complement activation [complement-dependent cytotoxicity (CDC)] or antibody-dependent cellular cytotoxicity (ADCC). Third, the antibody may have a specific effect on the tumor stroma, such as toxin delivery to the stromal cell or the blockade of angiogenesis by an antagonizing vasculature receptor or growth factor (24).

In case of a direct effect or effect on tumor stroma, it is important to consider that the efficacy of therapeutic mAbs is based on the target antigen, which, ideally, should be very abundant and specifically expressed on cancer cells or very selectively expressed in the tumor stroma. If possible, the antigen should be functionally related to the biology of cancer development and progression. In solid tumors, some of the most successful antibodies directly targeting tumor cells are those that block the ErbB family [which include epidermal growth factor receptor (EGFR) and human epidermal growth factor receptor 2 (HER2)] or vascular endothelial growth factor (VEGF). The anti HER2-specific antibody, trastuzumab, was the first specific antibody to be clinically approved by federal drug administration (FDA) in 1998 for the treatment of breast cancer HER2+ patients (25). In January 2008, FDA approval for trastuzumab was revised to include its usage as stand-alone treatment (without chemotherapy) in the adjuvant setting. As with prior approval, trastuzumab was limited to patients with HER2-positive breast cancer but could be prescribed to patients regardless of lymph node involvement. Following trastuzumab approval, also additional mAbs have shown encouraging results in the treatment of solid tumors. In colon cancer, the EGFR-specific antibody cetuximab has been shown to improve response and survival in patients and has been now indicated as first line of treatment of metastatic disease in combination with chemotherapy (26). Also in breast cancer, studies evaluating the effect of cetuximab in combination with cisplatin showed an ORR of 20% (versus 10% for cisplatin alone) (27). A similar positive activity has been also reported when cetuximab is used in combination with carboplatin (28). Several mAbs have also been approved for the treatment of hematological malignancies. The most well known and widely used of these is rituximab, which is directed against the CD20 receptor and has shown considerable success in patients with B cell non-Hodgkin’s lymphoma (NHL) and chronic lymphocytic leukemia (29). A major advance in this field of anti-CD20 mAbs is the advent of glyco-engineered antibodies with higher avidity for the CD16 receptor that ignites ADCC on NK cells. In addition, antibody-conjugated drugs or toxins have been approved by FDA. These include brentuximab vedotin for patients with CD30-positive Hodgkin’s lymphoma (30), which provided the first proof-in-principle that antibodies can selectively deliver active drug to cancer cells.

Antibodies have also been devised to enhance cellular immune responses by activating or antagonizing immunological receptors important for the regulation of anti-cancer immunity. These are thus referred to as immunomodulatory or immunostimulatory mAbs. The concept behind the usage of the immunomodulatory mAbs is based on the knowledge that the immune system, and in particular T lymphocyte activity, is regulated by a balance of co-stimulatory and co-inhibitory signals known as immune checkpoints. Under physiological conditions, these immune checkpoints are crucial to avoid autoimmunity and to protect bystander tissue during immune responses to infection. However, during cancer development, the balance conceivably shifts toward a reduced immune response, thereby resulting in unchecked tumor progression. The two molecules involved in the immune checkpoint regulation that have been most actively studied in the context of cancer immunotherapy are the CTLA-4 and PD-1 receptors. These are believed to inhibit immune responses at different levels and by different mechanisms (31).

The CTLA-4 receptor antagonizes binding between the TCR co-stimulatory molecule CD28 and the ligand CD86, thereby mediating a down modulation of T cell activation (32). An mAb able to functionally block CTLA-4 was made by Allison and colleagues who, in 1996, used a preclinical model to show that a significant antitumor response without overt immune toxicity was achieved when mice were treated with anti CTLA-4 antibody (33). These preclinical findings encouraged the production and testing of two fully humanized CTLA-4 antibodies, ipilimumab and tremelimumab, which began clinical trials in 2000. Both antibodies produced clinical response in patients with metastatic melanoma. However, ipilimumab was more successful in development, being associated with a 17% ORR in patients with advanced melanoma (34, 35) and opening a window of hope for long-term survival in a percentage of these patients (36). These compelling evidences led to the FDA approval of ipilimumab for patients with advanced melanoma in 2010. Recently, to provide a more precise estimate of long-term survival observed for ipilimumab-treated patients with advanced melanoma, a pooled analysis of OS data from multiple studies was compiled. The analysis, performed on 1,861 patients with available follow-up of at least 3 years and up to 10 years, showed that after 3 years the survival reach a plateau with a rate survival equal to 20%. The plateau was independent of prior therapy or ipilimumab dose. These data supported the durability of long-term survival in ipilimumab-treated patients with advanced melanoma (37). The immunostimulatory mechanism of action of ipilimumab and the severe autoimmune phenotype of CTLA-4 knock-out mice suggested that immune-related adverse effects can occur. However, no correlation between efficacy and immune-related toxicity has been observed in ipilimumab-treated melanoma patients (38). Responses to ipilimumab have also been reported in patients with advanced uveal and mucosal melanoma, who generally have a poor prognosis and otherwise have limited treatment options (39, 40).

Following studies with antibodies direct against CTLA-4, generally considered the godfather of immune checkpoints, attention now is focused on the PD-1:PD-1 ligand (PD-L1) pathway. Under physiologic conditions, PD-1 interacts with PD-1 ligand 1 or 2 (PD-L1 and PD-L2) to limit and regulate T cell activity in peripheral tissues during inflammatory or autoimmune processes. This co-inhibitory system has probably evolved to minimize collateral damage to healthy tissue and non-infected cells during clearance of intracellular viral orbacterial infections. Several mAb anti-PD-1 and anti-PD-L1 mAb are now currently undergoing clinical trials with very successful and encouraging results.

In advanced melanoma, the anti-PD-1 drug pembrolizumab (formerly MK-3475) showed a high rate of sustained tumor regression with mainly grade 1 or 2 toxic effects in 135 patients, including some with previous disease progression on ipilimumab. Moreover, the response rate did not significantly differ between patients who had received prior ipilimumab treatment and those who had not (9). In September 2014, pembrolizumab (Keytruda) was the first approved anti-PD-1 drug by the FDA for the treatment of melanoma patients with relapsed or refractory disease. Pembrolizumab will be used in patients following treatment with ipilimumab, or after treatment with ipilimumab and a BRAF inhibitor in patients who carry mutated BRAF. A pivotal phase II study (KEYNOTE-002) has already shown that pembrolizumab improves the progression-free survival (PFS) compared to chemotherapy in patients with ipilimumab-refractory advanced melanoma (*n* = 540). At 6 months, the PFS rates for pembrolizumab-treated patients were more than 30% compared to 16% obtained with chemotherapy treatments (41). A study recently conducted on 834 patients with advance melanoma also showed that the usage of the anti-PD-1 antibody, pembrolizumab, prolonged PFS and OS and had less high-grade toxicity than did ipilimumab (42).

In parallel with pembrolizumab, other anti-PD-1 mAbs have been tested in clinical trials. Nivolumab, a fully human PD-1 blocking antibody, has been developed, and a durable clinical response (43, 44) together with increased survival has been reported in a large phase I trial recruiting patients with different tumor types, including melanoma, renal cell carcinoma, colorectal cancer, and non-small-cell lung cancer (NSCLC) (45). Significantly, increased survival was also observed by treating 418 previously untreated patients who had metastatic melanoma without a BRAF mutation with nivolumab versus dacarbazine. The results showed 72.9% overall rate of survival at 1 year in the nivolumab group compared with 42.1% in the dacarbazine group (46). A phase III study conducted also in melanoma also showed that nivolumab led to a greater proportion of patients achieving an objective response and fewer toxic effects than with alternative available chemotherapy regimens for patients with advanced melanoma that has progressed after ipilimumab or ipilimumab and a BRAF inhibitor (47). These encouraging results led to an accelerate approval in December 2014 of nivolumab by FDA for the treatment of metastatic melanoma. In March 2015, nivolumab received also approval by the FDA for the second line of treatment of metastatic squamous NSCLC patients.

Potential predictive biomarkers for PD-1/PD-L1 targeting treatment include PD-L1 expression by tumor cells by a variety of IHC techniques and laboratory assays. Some studies have suggested that the response to nivolumab might correlate with the expression of PD-L1 (48). Indeed, by performing preliminary analysis on 42 patients with different cancer types before their treatment with nivolumab, it has been identified that PD-L1 expression on tumor cell surface as one factor associated with the clinical activity of anti-PD-1 therapy (43).

Despite these very encouraging results, the observation that a significant number of patients with PD-L1 positive expression (PD-L1+) tumors still do not respond to PD-1 pathway blockade (43) or still lack of increased OS compared to patients with negative PD-L1 expression (46) suggest that other factors might be needed to ensure an effective response to nivolumab therapy. Thus, additional studies might be necessary to select patients with increased likelihood to respond to nivolumab and to other PD-1/PD-L1 targeting treatments and to possible co-target factors in combination treatment regimens to ensure a more successful response.

On April 2015, nivolumab was approved from European Medicines Agency (EMA) for the treatment of advanced melanoma in first line. On May 2015, EMA approved nivolumab for the treatment of NSCLC in second line and pembrolizumab had the same indication of nivolumab in first line.

## Cancer Vaccines

Among the different immunotherapeutic strategies that have been introduced, cancer vaccination is the one most often investigated in a variety of preclinical and clinical settings (49). Since the first recognition of shared tumor-associated antigens in melanoma 24 years ago (50), the appeal of potential vaccine that triggers specific antitumor immune response has led to considerable research in this area. However, most clinical trials have yielded disappointing results. In melanoma, the usage of gp100, a melanosomal protein, has been studied in several trials. The vaccination with an optimized peptide derived from melanoma-associated antigen gp100 used in association with ipilimumab did not show any encouraging results compared with ipilimumab alone (36). However, gp100 vaccination applied concomitantly with IL-2 showed significantly improved PFS in a cohort of patients with metastatic melanoma (51). Another successful vaccine showing efficacy in large controlled phase III studies was sipuleucel-T, which consists of a preparation of autologous antigen-presenting cells activated and pulsed with a fusion protein consisting of prostatic acid phosphate (PAP) and granulocyte-macrophage colony-stimulating factor (GM-CSF). Treatment with sipuleucel-T in patients with metastatic prostate cancer improved median survival by approximately 4 months. Currently, sipuleucel-T is the only cancer vaccine approved by the FDA (52). This complex cell product, which involves three sequential leukapheresis leukocyte suspensions being cultured for 48 h with the PAP–GM-CSF fusion protein, contains both antigen-presenting cells and activated T cells and thus the exact mechanism of action is undefined. However, its use has been shown to prolong OS without improving PFS. A phase III trial utilizing this approach in melanoma yielded disappointing results (53).

In order to understand the problems associated with the lack of success of vaccine in cancer treatment, it is important to consider that the therapeutic vaccination against cancer is mostly based on the idea that there is a repertoire of functionally competent effector and memory T cells with specificity for tumor cell antigens at sufficient frequencies to control tumor progression (49). However, at least three major hurdles need to be considered. First, tumor antigens used in vaccination are self-proteins and T cells with high affinity receptors recognizing these antigens might have been deleted through thymic and peripheral negative selection. Second, tumors deploy panoply of molecules that are locally and systemically immunosuppressive, meaning mechanisms of immune tolerance against cancer-associated antigens need to be overcome. Lastly, tumor cells are notoriously heterogeneous and their antigen expression of a given tumor can range from completely negative to highly expressed changing also upon microenvironment and systemic conditions. Hence, it should be considered that cancer vaccines not only induce a newly primed T cell repertoire *de novo* but may also need to reactivate and potentially reeducate pre-existing tumor-reactive memory T cells. As such, the presence or absence of pre-existing effector/memory T cell responses represents an important prognostic biomarker for response to vaccination.

In addition, in order to have a successful vaccination, a high quality CD4 and CD8 effector and memory T cell responses against tumor-associated antigens need to be achieved.

Least but not last, the strength and type of the vaccine-induced immune response are truly determined by the amount of antigen presented by the activation/maturation of dendritic cells induced through the stimulation of danger-associated molecular pattern (DAMP) receptors (49).

A cumbersome but actively tested approach is to culture patients’ dendritic cells *ex vivo*, after loading their antigen-presentation pathways with sources of tumor antigens before being reinfused to the patient (54). Such complex and invividualized approach is difficult to test in large-scale randomized trials. However, the use of dendritic cells differentiated in culture with monocytes and loaded with whole cell tumor lysates is being tested in phase III trials in glioblastoma to prevent postsurgical relapse as well as in melanoma.

Nowadays, the emphasis of research is placed in vaccinating against the individual neoantigens of each malignancy. These are derived from the non-synonymous mutations encoded in the mutatome of each malignant disease case. A variety of research lines are suggesting the superiority of such individual antigens over shared antigens. However, the necessary biotechnology and bioinformatics for epitope prediction and individualized vaccine production are only at their infancy to consider wide scale deployment (55, 56). Poly-peptide and mRNA vaccines containing the mutations giving rise to HLA class I and II antigenic determinants are the most promising approaches.

In summary, despite mostly negative results, there is a continuous interest in vaccine immunotherapy. The optimization and the refinement of existing and the development of novel vaccines, the identification of potential predictive biomarkers, and the combinations with other forms of immunotherapy are goals that continue to be actively pursued.

## Combinatorial Immunotherapy

Optimizing immunotherapy can require treatments affecting multiple and combinatorial aspects of the immune response (57, 58). One immunotherapy combination consists of the use of cancer vaccines to generate anti-tumor T cells and immune checkpoints blockade to prevent T cell anergy. Survival studies performed in the B16 melanoma and CT26 colon carcinoma tumor models showed that the combination of PD-1 blockade with GM-CSF-secreting tumor cell immunotherapy leads to improved antitumor responses by augmenting the tumor-reactive T-cell responses induced by the cellular immunotherapy (59). In another preclinical study, the combination of CTLA-4 and PD-1 blockade was found more than twice as effective as either alone in promoting the rejection of B16 melanomas. The results suggested that the combination blockade of the PD-1/PD-L1- and CTLA-4-negative co-stimulatory pathways allowed tumor-specific T cells that would otherwise be inactivated to continue to expand and carry out effector functions, thereby shifting the tumor microenvironment from suppressive to inflammatory (60). Prompted by these studies, results of combinatorial immunotherapy have been reported also in humans. First, a small trial showed that periodic infusions of anti-CTLA-4 antibodies after vaccination with irradiated, autologous tumor cells engineered to secrete GM-CSF (GVAX) generated clinically meaningful antitumor immunity in a majority of metastatic melanoma patients (61). Similarly, in a phase II trial, 245 patients with melanoma were randomized to receive ipilimumab and GM-CSF in combination or ipilimumab alone (60). The survival rate after 1 year of treatment in the combination arm was 68.9% compared with 52.9% in the monotherapy arm, while the median OS in the combination arm was 17.5 months compared with 12.7 months in the group of patients that received ipilimumab alone. Interestingly, GM-CSF mitigates the immune-related adverse events of ipilimumab. Most excitingly, the combination of ipilimumab with bevacizumab (anti VEGF) in a phase I study of 22 melanoma patients has shown impressive results with 1-year survival rate of 72% (53). Another promising combination approach of bevacizumab plus MPDL3280A (an anti-PD-L1) is currently being evaluated (NCT01633970).

Encouraging evidences also derived from a study performed on 86 patients with advanced melanoma treated with nivolumab and ipilimumab. The results showed that combinatorial therapy with nivolumab and ipilimumab induced a rapid and deep tumor regression in a substantial proportion of patients (62). Similar evidences have been also recently shown in a study involving 142 patients with metastatic melanoma who had not previously received treatment (63). The results demonstrated greater objective-response rate and PFS among patients treated with nivolumab combined with ipilimumab than with ipilimumab monotherapy. Taken altogether, these observations showed that the usage in combinations of immunomodulatory approaches holds an absolutely unprecedented hope for a robust impact on the survival of cancer patients and may represent a decisive turning point for cancer therapy.

## Conclusion

The immunotherapy field has exploded with the recent advances, especially in melanoma, demonstrating an improvement in survival and a durability of response. However, the perception is that this is not limited to any particular malignant disease but will be widely used across indications in oncology and hematology. The field is now investigating the role of novel approaches to be used either alone or in combination, such as (1) the blockade of lymphocyte activation gene 3 (LAG3), which normally stimulates Treg cells and inhibits CD8+ effector T cells through its interaction with MHC class II molecules and (2) T-cell membrane protein 3 (TIM3) antagonists, which have been shown to increase antitumor T cell responses when used in combination with anti-PD-1 agents. Immunostimulatory monoclonal antibodies that act as agonist of activatory receptors on immune system cells are also under fast clinical development (anti-OX40, anti-CD137, anti-CD27, anti-GITR, anti-CD40 mAbs).

Immunotherapy now offers treatments with the potential for long-term cure. Given these promising results and the large number of trials currently underway, it is very tempting to anticipate a bright future, especially for the treatment of advanced melanoma.

## Author Contributions

MLA, IM, and PAA wrote and approved the manuscript.

## Conflict of Interest Statement

Maria Libera Ascierto has no conflict of interest to declare. Ignacio Melero has an advisory role for Bristol Myers Squibb, Roche-Genentech, AstraZeneca, Miltenyi biotec, Leadartis, Boehringer Ingelheim. He received research funds from Bristol Meyer Squibb, Roche-Genentech, and Ventana from Pfizer and Boehringer Ingelheim. Paolo Antonio Ascierto has a consultant/advisory role for Bristol Meyer Squibb, Roche-Genentech, Merck Sharp and Dohme (MSD), GlaxoSmithKline (GSK), Novartis, Ventana, and Amgen. He received research funds from Bristol Meyer Squibb, Roche-Genentech, and Ventana.
